# Clinical application of a three-dimensional-printed model in the treatment of intracranial and extracranial communicating tumors: a pilot study

**DOI:** 10.1186/s41205-024-00202-5

**Published:** 2024-01-22

**Authors:** Xiang-heng Zhang, Jiahao Li, Zhenqiang He, Dikan Wang, Guiqing Liao, Si-en Zhang, Hao Duan, Yonggao Mou, Yujie Liang

**Affiliations:** 1 Department of Neurosurgery, State Key Laboratory of Oncology in South China, Collaborative Innovation Center for Cancer Medicine, Sun Yat-Sen University Cancer Center, Guangzhou, China; 2 Department of Oral and Maxillofacial Surgery, Hospital of Stomatology, Guanghua School of Stomatology, Sun Yat-Sen University, Guangdong Provincial Key Laboratory of Stomatology, Guangzhou, China

**Keywords:** 3D printing, Intracranial and extracranial communicating tumor, Surgical planning, Resident education

## Abstract

**Background:**

Surgical management for intracranial and extracranial communicating tumors is difficult due to the complex anatomical structures. Therefore, assisting methods are urgently needed. Accordingly, this study aimed to investigate the utility of a three-dimensional (3D)-printed model in the treatment of intracranial and extracranial communicating tumors as well as its applicability in surgical planning and resident education.

**Methods:**

Individualized 3D-printed models were created for eight patients with intracranial and extracranial communicating tumors. Based on these 3D-printed models, a comprehensive surgical plan was made for each patient, after which the patients underwent surgery. The clinicopathological data of patients were collected and retrospectively analyzed to determine surgical outcomes. To examine the educational capability of the 3D-printed models, specialists and resident doctors were invited to review three of these cases and then rate the clinical utility of the models using a questionnaire.

**Results:**

The 3D-printed models accurately replicated anatomical structures, including the tumor, surrounding structures, and the skull. Based on these models, customized surgical approaches, including the orbitozygomatic approach and transcervical approach, were designed for the patients. Although parameters such as operation time and blood loss varied among the patients, satisfactory surgical outcomes were achieved, with only one patient developing a postoperative complication. Regarding the educational applicability of the 3D-printed model, the mean agreement for all eight questionnaire items was above six (seven being complete agreement). Moreover, no significant difference was noted in the agreement scores between specialists and residents.

**Conclusion:**

The results revealed that 3D-printed models have good structural accuracy and are potentially beneficial in developing surgical approaches and educating residents. Further research is needed to test the true applicability of these models in the treatment of intracranial and extracranial communicating tumors.

**Supplementary Information:**

The online version contains supplementary material available at 10.1186/s41205-024-00202-5.

## Background

Intracranial and extracranial communicating tumor, also referred to as intra-and-extracranial tumor, is a distinct type of skull base tumor. Currently, there exists no classification system to categorize such tumors into their subtypes. However, according to their anatomical position, they can be roughly divided into tumors of the anterior skull base, tumors of the middle skull base, and tumors of the posterior skull base [1]. All three types manifest as both malignant and benign tumors [2]. Among primary skull base tumors, meningioma is the most prevalent type, with an incidence of 2 per 100,000 people per year. However, skull base metastatic tumors are more common with an incidence of 18 per 100,000 people per year [3]. Until the late twentieth century, intracranial and extracranial communicating tumors were considered inoperable owing to the pleomorphism and involvement of several complex anatomical structures, such as the cranial nerves, internal carotid artery, and temporomandibular joint. However, advancements in technology that aid surgery, such as neuronavigation and high resolution magnetic resonance imaging (MRI), have revolutionized the treatment from partial to gross total resection [4]. In addition, for malignancies of the craniomaxillofacial region, adjuvant radiotherapy and chemotherapy are often mandatory to maximize clinical outcomes [5–7]. Irrespective of the availability of state-of-the-art techniques, surgical management can still be challenging [8]. Moreover, the risk of complications following surgery remains high, and the treatment is usually multi-disciplinary, involving experienced neurosurgeons, oral-maxillofacial surgeons, otolaryngologists, and ophthalmologists.

Three-dimensional (3D) printing, also known as additive manufacturing, is being increasingly used in health care. The earliest 3D printing technology was stereolithography (SLA) invented by Charles W. Hull in the 1980s, which was believed to revolutionize research and hands-on medical learning [9]. In 1998, the invention of PolyJet technology raised the accuracy of 3D printing [10]. In the current surgical scenario, 3D printing is mainly used to fabricate surgical models to visualize complex structures and guide operations. In addition, it has demonstrated applicability in fabricating implants for use in osseous operations [11]. As 3D-printed models accurately replicate inter-structural relationships, they are helping beginners learn anatomy better and assisting surgeons in surgical decision-making. The efficacy of 3D models printed using the PolyJet technology have been confirmed by several studies [12–14]. However, there is no report assessing the utility of 3D-printed models in the treatment of intracranial and extracranial communicating tumors.

This retrospective study reviewed patients with intracranial and extracranial communicating tumors for whom 3D-printed models were used to aid surgical planning. In addition, the primary surgical procedures adopted and characteristics of surgical approaches were summarized. Moreover, oral-maxillofacial specialists and resident doctors were invited to complete a questionnaire to evaluate the applicability of 3D-printed models in resident education.

## Methods

### Study population

This study was conducted at the Sun Yat-Sen University Cancer Center from 1 August 2016 to 30 October 2020. A total of nine patients, for whom 3D-printed models were fabricated, were recruited on the following inclusion criteria: 1) radiological confirmation of intracranial and extracranial communication; 2) pathological confirmation of tumor (benign or malignant); and 3) availability of complete clinical data. Of these, one patient was excluded owing to the presence of a recurrent tumor, for which radio-chemotherapy was recommended rather than surgery. The remaining eight patients (Table 1) underwent combined neurosurgery and oral-maxillofacial surgery.

**Table 1:** Summary of cases

Patient	Gender	Age (Years)	Location of tumor	Tumor size (mm)	Pathological diagnosis
1	Female	61	Right middle cranial fossa, sphenoid bone and infratemporal fossa	56*50	Adenoid cystic carcinoma
2	Male	44	Left middle cranial fossa, zygomatic arch and temporal bone	52*45	Giant cell tumor of bone
3	Female	49	Right posterior cranial fossa and neck	33*28	Paraganglioma
4	Male	29	Right middle cranial fossa, sphenoid bone and infratemporal fossa	48*55	Fibrosarcoma
5	Male	33	Left middle cranial fossa, temporal bone and infratemporal fossa	46*64	Meningioma
6	Female	78	Right anterior-middle cranial fossa and sphenoid bone	75*57	Atypical Meningioma
7	Female	32	Left middle cranial fossa and infratemporal fossa	43*40	Giant cell tumor of tendon sheath
8	Female	46	Left middle cranial fossa and infratemporal fossa	53*32	Trigeminal nerve schwannoma

### Data collection

Using the hospital’s electronic medical record system, demographic and clinicopathological data were collected, including sex, age, tumor site, tumor size, radiological images (MRI or computed tomography angiography (CTA)) of the tumor at its widest diameter and at the intracranial and extracranial communication point, time of operation, length of operation, blood loss, length of stays, pathological diagnosis, and post-operative complication. The size of tumor was defined as the largest diameter of the tumor observed on enhanced T1-weighted MRI scans obtained before treatment. In addition, data on postoperative complications, including infection, facial paralysis and loss of eyesight were recorded.

### 3D model printing

The printing procedure has been thoroughly described in our previous study [15]. Briefly, the Mimics 3D image reconstruction software (Materialise, Leuven, Belgium) was used to process the data obtained from CT, MRI, and CTA scans to create a 3D reconstruction of the craniofacial strutures. The reconstruction was then transformed into a STL file. Objet350 Connex3 printer (Stratasys, Rehovot, Israel) was used in this study. Then, the STL files were loaded onto the printer, and parameters such as texture, color, and transparency were set using the Objet Studio software (Ver. 9.2.11.6817). Printing materials included Support Fullcure 705, VeroWhitePlus Fullcure 835, Veroclear Fullcure 810, Objet RGD836 Vero Yellow, Objet RGD851 Vero Magenta, and Objet RGD843 VeroCyan. The printer sprayed the materials onto the build tray, and every layer of materials was cured by UV light. After printing, the model was visually cross-checked with radiological scans.

### Model application

Three out of the eight 3D-printed models were preserved in our department, which were evaluated by eight oral-maxillofacial specialists and 25 resident doctors. In addition, doctors were provided access to the three patients’ basic information (e.g., age, sex, and preliminary diagnosis), medical history (e.g., chief compliant, and history of present illness), physical examinations, and radiological imaging (MRI, CT, or CTA). Next, doctors were invited to watch a short movie explaining the anatomical structure of each 3D-printed model (three movies in total including Movie 1–2) and then examine corresponding surgical approaches. Finally, the doctors were invited to complete a questionnaire based on Zhang et al.’s work [16] (as seen in Table 2) regarding the applicability of 3D-printed model in clinical scenarios, both as an auxiliary technique to aid surgery and as a training medium to educate resident doctors.

**Table 2:** Applicability of 3D-printed models in clinical circumstances

Questions	Mean agreement ± SD^a^			*P* value
	All (*n* = 33)	Specialists (*n* = 8)	Residents (*n* = 25)	
The model accurately restored the anatomical structure of the skull base, the tumor and surrounding tissue	6.06 ± 0.93	5.63 ± 1.19	6.20 ± 0.82	0.20
Compared with MRI scanning, the 3D-printed model is better to provide information pertaining to the three-dimensional spatial structure of the skull base, the tumor and surrounding tissue	6.09 ± 1.13	6.13 ± 1.46	6.08 ± 1.04	0.62
The model is of great value to help learn the anatomical knowledge of the skull base	6.82 ± 0.58	7.00 ± 0.00	6.76 ± 0.66	0.55
The model should be used as a teaching prop in clinical circumstances	6.67 ± 0.65	6.88 ± 0.35	6.60 ± 0.71	0.37
The model is of value for surgeons to understand potential surgical risk and develop surgical planning	6.27 ± 1.21	6.63 ± 0.52	6.16 ± 1.34	0.67
The 3D printing model can help surgeons improve the surgical efficiency and confidence	6.12 ± 1.32	6.50 ± 0.53	6.00 ± 1.47	0.74
In complicated cases, the model can be used as a supplementary assisting method to MRI to overcome limitations of MRI scanning of intracranial and extracranial communicating tumors	6.30 ± 1.21	6.75 ± 0.46	6.16 ± 1.34	0.43
The model is helpful for doctors to communicate with patients prior to surgery (to help patients understand their diseases)	6.70 ± 0.81	7.00 ± 0.00	6.60 ± 0.91	0.30

^a^0 = completely disagree, 7 = completely agree

### Statistical analysis

All statistical analyses were conducted using SPSS software, version 25 (IBM Corp., Armonk, NY, USA). Differences between specialists and residents’ agreement were calculated using Wilcoxon rank sum test. *P* value < 0.05 was considered statistically significant.

## Results

### Patient demographic characteristics

Eight patients who were diagnosed with intracranial and extracranial communicating tumor (Table 1 and Table S1) were included in this study. Among these patients, five (62.5%) were female and three (37.5%) were male, with a mean age of 46.5 (range: 29–78) years. Seven (87.5%) patients had tumors in the middle cranial fossa, with one (12.5%) having additional involvement in the anterior cranial fossa. The remaining patient (12.5%) had tumor involvement in the posterior cranial fossa. Preoperative radiological scans demonstrated that in six (75%) patients, the widest tumor diameter was > 5 cm. The most common pathological type of neoplasm was meningioma (two cases, 25%). Other pathological types included adenoid cystic carcinoma, giant cell tumor of bone, giant cell tumor of tendon sheath, paraganglioma, fibrosarcoma and trigeminal nerve schwannoma. The mean operation time was 7.55 (range: 3.50–12.75) h, and the mean blood loss was 750 (range: 100–2800) mL. The mean length of stays was 25 (range: 20–35) days. Among all patients, only one (12.5%) developed a postoperative complication (loss of eyesight).

### Development of accurate 3D-printed models

**Fig. 1:**
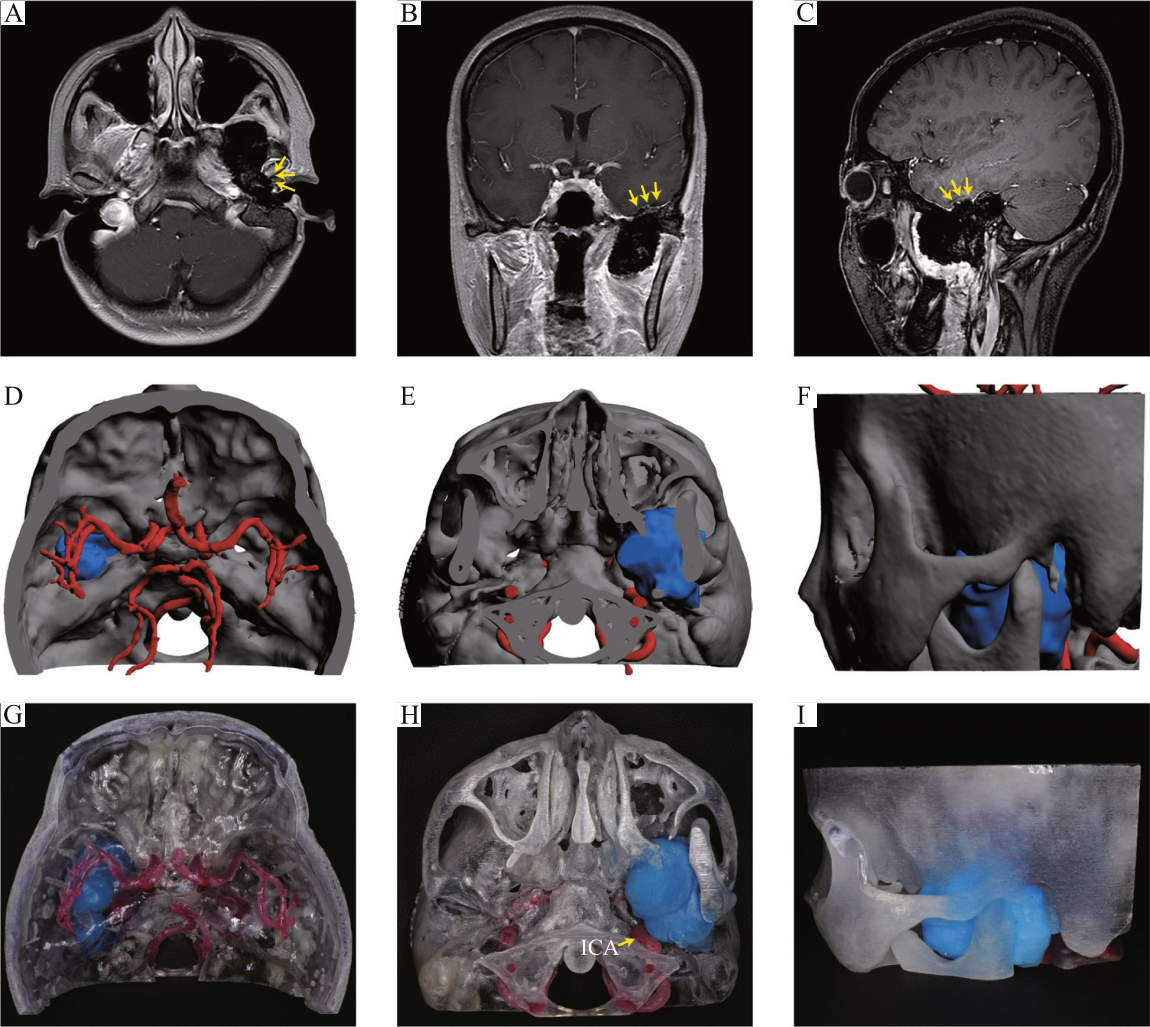
Demonstration of case seven. **A** The tumor can be seen partially invading the left condyle (yellow arrows). **B** Left temporal lobe can be seen pushed upwards (yellow arrows). **C** Inconsistency in the dura can be observed (yellow arrows). **D**-**F** 3D reconstruction of the anatomical structure. **G**-**I** Different views of the 3D-printed model showing that the internal carotid artery (yellow arrow) was neither invaded nor encircled by the tumor. Abbreviation: ICA (internal carotid artery)

Based on the aforementioned printing method, accurate 3D-printed models of intracranial and extracranial communicating tumors, including their surrounding structures, were created for all eight patients. An example of the 3D-printed model (case seven) is shown in Fig. 1, with T1-weighted MRI scans demonstrating the low signal mass in the left infratemporal fossa that partially invaded the mandibular condyle (Fig. 1A). The scans also demonstrated the left temporal lobe that was pushed upwards (Fig. 1B) along with inconsistency in the dura (Fig. 1C). Using data from MRI, CT, and CTA scans, a virtual 3D-printed model was constructed (Fig. 1D, E), which was loaded onto the software for model printing. To demonstrate the tumor’s relative position, the skull was also 3D-printed, with a half-open design, making it easy to visualize tumors from all angles. Moreover, the tumor was marked with a distinct color to distinguish it from the surrounding tissue. In addition, a transparent printing material was selected to create a “see-through” effect to allow resident doctors to easily locate the tumor (Fig. 1G-I and Movie 1).

The printed models accurately replicated anatomical structures and demonstrated whether important structures, such as blood vessels (e.g., internal carotid artery and circle of Willis) or nerves (e.g., trigeminal nerve and optic nerve) were pushed or invaded (Fig. S1-S2).

### Comprehensive surgical approach

**Fig. 2:**
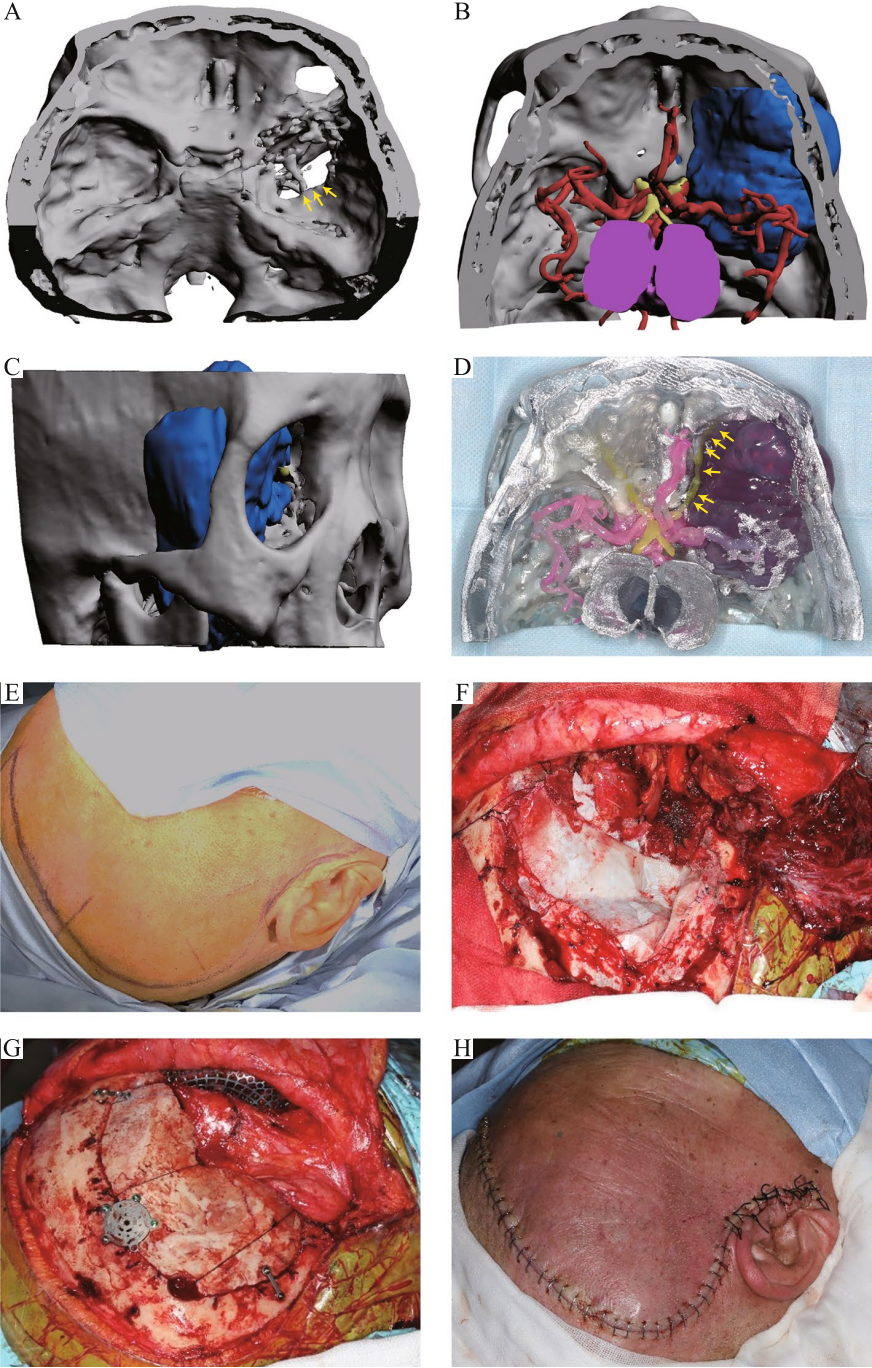
The standard orbitozygomatic approach for intracranial and extracranial communicating tumors in the middle skull base. **A**-**C** The 3D reconstruction showing the anterior and middle skull base penetration and invasion of the right greater wing of sphenoid (yellow arrows) and orbit by the tumor. **D** The right optic nerve can be seen segmentally embedded in the tumor (yellow arrows). **E**-**H** Basic steps involved in the orbitozygomatic approach

Case six is a typical example to demonstrate the adopted surgical approach in intracranial and extracranial communicating tumor of the middle skull base. The patient visited us with the chief complaint of deteriorating eyesight in the right eye and numbness in the right cheek for eight months. A preliminary examination at admission revealed signs of diplopia. A subsequent 3D reconstruction showed that the tumor had penetrated the anterior and middle skull base and had invaded the right greater wing of the sphenoid bone (Fig. 2A, B). Moreover, the intracranial portion of the tumor partially occupied the right anterior and middle cranial fossa; in contrast, the extracranial portion partially occupied the right orbit and right infratemporal fossa (Fig. 2B, C). Additionally, the 3D-printed model revealed that the tumor had invaded the right optic nerve (Fig. 2D), which was consistent with clinical symptoms. Considering that the tumor was large and had partially encased the right optic nerve, an endoscopic endonasal approach was not considered as it rarely results in maximal resection. Instead, an open, two-step orbitozygomatic approach was adopted, with the first step targeting the extracranial portion of the tumor and the second targeting the intracranial portion. Initially, a frontotemporal incision was made to access the middle cranial fossa (Fig. 2E) by reflecting the skin flap forward. Then, the temporalis was also incised and reflected forward to reveal the zygoma. Following that, the zygoma was incised to reveal the anterolateral portion of the tumor in the infratemporal fossa. The bony tissue surrounding the oval foramen was then partially removed to expose the communication point (Fig. 2F). Subsequently, a frontotemporal bone flap was harvested to reveal the intracranial portion of the tumor. Then, using an operating microscope, the tumor was removed along with tumor-involved bony tissue and dura. Since the intraoperative frozen-section pathology revealed atypical meningioma, the right optic nerve was sacrificed to improve the prognosis. Finally, the right temporalis myofascial flap was used to reconstruct the skull base defect, and the wound was closed layer by layer (Fig. 2G, H). After the operation, the patient developed complete vision loss in the right eye.

**Fig. 3:**
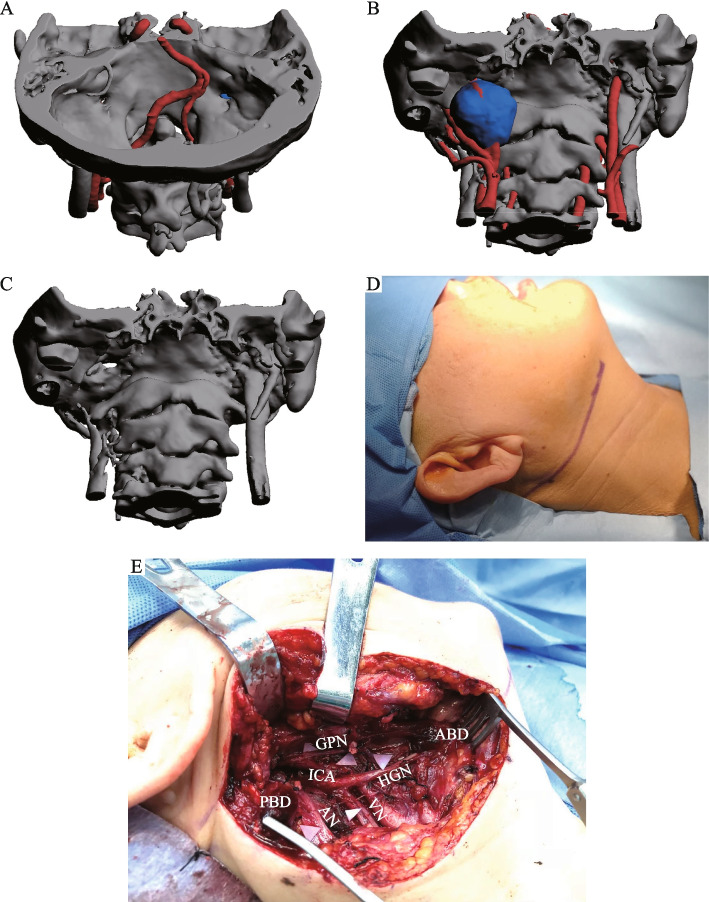
The surgical approach adopted for intracranial and extracranial communicating tumors in the posterior skull base. **A**-**C** Screenshots of 3D reconstruction PDF files. **D**, **E** Basic surgical steps. Abbreviations: GPN (glossopharyngeal nerve), HGN (hypoglossal nerve), VN (vagus nerve), AN (accessory nerve), ABD (anterior belly of digastric muscle) and PBD (posterior belly of digastric muscle)

In the patient with involvement of the posterior skull base (case three), the tumor had invaded the jugular foramen and did not fully extend into the cranial cavity (Fig. 3A-C). As a result, a more conservative transcervical approach was utilized for this case. First, a submandibular incision was made to reveal deep tissue of the superficial cervical fascia (Fig. 3D). Then, the facial artery and vein were identified and ligated, while the marginal mandibular branch of the facial nerve was preserved. Subsequently, the mandible was pulled outwards to reveal the infratemporal fossa, then the posterior belly of digastric muscle was dissected and external carotid artery was ligated. Following full exposure of the cervical deep tissue (Fig. 3E), the accessory nerve was traced back to the tumor using a microscope. The tumor was then completely resected and separated from the internal jugular vein. Finally, the skull base defect was repaired by posterior belly of digastric muscle and partial-thickness sternocleidomastoid muscle flap.

### Improvement in resident education

Regarding the applicability of 3D-printed model in education, the mean agreement score for all eight questions on was above six (seven being complete agreement). The lowest mean agreement score was documented for the question on the model’s accuracy (question one): 12 doctors (36.36%) rated this parameter seven, 13 (39.39%) rated six, seven (21.21%) rated five and one (3.03%) rated three. Among specialists, the mean score for this parameter was also the lowest. The highest variability was noted for the 3D-printed model’s utility to improve surgical efficiency and confidence (question six), whereas the highest agreement was recorded for the model’s ability to help learn anatomy (question three).

The lowest mean agreement in specialists from question one and the highest variability in all doctors from question six possibly originated from the varied level of clinical skills and understanding of surgical operation. Moreover, although no significant difference was found in the agreement scores between specialists and residents, in the 200 answers provided by 25 residents, only seven (3.5%) were below the score of four and an even lower percentage (3.13% of 64 answers) was found in specialists, suggesting an overall good acceptance of the 3D-printed model.

## Discussion

Surgery is the mainstay treatment for intracranial and extracranial communicating tumors. However, surgical management is still considered a challenge as these tumors are often in proximity to important structures in the cranio-maxillofacial region [4]. 3D printing, as an emerging technology, is being used to fabricate anatomical models. However, no report has specifically targeted its use in the surgical treatment of intracranial and extracranial communicating tumors. To the best of our knowledge, this is the first study to use a 3D-printed model in the treatment of intracranial and extracranial communicating tumors that achieved promising results.

Briefly, individualized 3D-printed tumor models were fabricated for eight patients and were later used for customizing surgical approaches and for resident education. Of all patients, seven had tumor involvement in the middle cranial fossa (including one case with anterior cranial fossa involvement) and one had tumor involvement in the posterior cranial fossa. The higher proportion of patients with tumors in the middle cranial fossa may be partially attributed to the fact that most skull base tumors requiring oral-maxillofacial surgery are located in this region [17]. Moreover, six out of eight patients had a tumor with the largest diameter being > 5 cm, which corresponds to the insidious growth pattern observed in ventral skull base tumors [18]. And since intracranial and extracranial communicating tumors include a wide range of pathological types, in this study, only two cases had the same pathological diagnosis (meningioma).

Particularly, patient six developed loss of eyesight as a postoperative complication; however, it must be noted that the patient was already showing signs of diplopia at admission. In addition, MRI scans and the 3D-printed model confirmed that the right optic nerve was segmentally embedded in the tumor. Moreover, for this patent, intraoperative frozen-section pathology revealed atypical meningioma, which shows high invasiveness and thus required total resection to promote prognosis [19]. Owing to all these factors, the affected optic nerve was intentionally removed, which resulted in the loss of eyesight.

The surgical approaches for intracranial and extracranial communicating tumors need to be extremely precise and mainly depend on the following factors: 1) tumor size; 2) location; 3) extension; and 4) operator’s preference. The common approaches for anterior skull base tumors include unilateral frontal, bilateral frontal, modified orbitozygomatic and pterional approach [6]. For middle skull base tumors, the transmandibular, transmaxilliary and transcervical approaches are widely used [17]. However, once the tumor breaks through the skull base into the cranial cavity, surgical management often requires focusing on both the intracranial and extracranial lesions. In addition, the craniomaxillofacial region is abundant in vasculature and nerves, which requires careful handling to avoid surgical complications such as embolic stroke, venous infection with cascading ischemia of brain tissue, diplopia or loss of eyesight [20]. Moreover, relying on the pathway through which the tumor travels into the cranial cavity is often inadequate for complete resection. Therefore, a simple one-way surgical approach is far from satisfactory for such tumors. In the present study, only one patient (case six) presented with anterior skull base involvement, with the majority of intracranial tumor mass remaining in the middle cranial fossa. Notably, the tumor had additionally broken through the lateral orbital wall. As a result, a typical orbitozygomatic approach was adopted for this patient. In this approach, the zygomatic arch is sectioned and then the temporal muscle is reflected. Following this, frontotemporosphenoidal craniotomy is performed. This approach provides optimal exposure both horizontally and vertically [21]. In contrast, the standard pterional approach involves harvesting a smaller frontotemporal bone flap, without performing zygomatic osteotomy. This approach mainly aims at accessing an area that is sufficient to operate microscopically [22]. Thus, it can be concluded that without zygomatic osteotomy, it would have been difficult to completely resect the tumor within the infratemporal fossa that invaded muscle, bone, and other soft tissue. Therefore, the orbitozygomatic approach was chosen with modifications based on tumor invasion to adequately expose the tumor in the infratemporal fossa. This approach additionally assisted in ensuring minimum bone defect and improving the postoperative quality of life for patients.

The lateral skull base approach and the infratemporal fossa approach are often used to resect paraganglioma [23], as these tumors might invade the cavernous sinus through the jugular foramen [6]. In the present study, only one patient (case three) had tumor involvement in the posterior skull base. However, neither of these two approaches was adopted, primarily because the size of the intracranial portion of the tumor was rather small, which allowed its extraction using an operating microscope. Notably, in this approach, it is crucial to protect cervical nerves, including glossopharyngeal nerve, hypoglossal nerve, accessory nerve, vagus nerve and accompanying cervical blood vessels, especially internal carotid artery. This can be achieved by ensuring the following: a) familiarity of the surgeon with cervical anatomy; b) optimal exposure to the operating area, which requires assistant’s constant focus and change in the angle of surgical hook, if required; and c) careful separation of cervical nerves from their surrounding structures.

Currently, two-dimensional black-and-white scans remain the standard tool to educate patients during preoperative doctor-patient communication [24]. However, restricted by the inadequate presentation of the disease by these scans, patients can hardly comprehend the anatomy and accompanying risks of complications. A few studies implemented 3D-printed models during surgical treatment and have demonstrated promising results [25]. In our previous study [15], 3D-printed models were used to pre-operatively educate patients with skull base meningioma, which improved doctor-patient communication and patients’ understanding of the disease. In the present study, we focused on the utility of these models in resident education.

Several studies have demonstrated the utility of 3D-printed models in surgical training [26, 27]. In the current study, eight oral-maxillofacial specialists and 25 resident doctors were invited to evaluate the applicability of 3D-printed models in surgical treatment. It was found that most doctors agreed with the utility of these models both in treatment and education. Moreover, there was high agreement among all doctors regarding the responses to the eight questions, and there were no significant differences between specialists’ and residents’ responses. This indicates that using 3D-printed models is equally useful to specialists and residents, which also indicates its potential in the field of surgery.

In recent years, a similar technology, multimodal image fusion (MIF), has also been proposed to assist surgery. Jian et al. evaluated the usefulness of MIF in a total of 47 skull base tumor cases and acknowledged the outstanding performance of MIF [28]. Despite the towering case number, in this study, assessment of the fused 3D images was conducted by only one person, the operating surgeon, which undermines the credibility of the research. In their previous study, MIF was used to locate the offending vessel of compressed nerves, which helped the surgeons visualize the spatial relationship preoperatively [29]. They also combined MIF with dynamic CT for evaluation of percutaneous balloon compression surgery, concluding that MIF was helpful [30]. Through the series of research, it is clear that 3D printing and MIF are both capable of demonstrating spatial information, and considering the printing errors, MIF might even be more advantageous in this aspect. MIF also spares patients the extra costs for the printing of a 3D model. However, 3D printing maybe more practical when used as a teaching prop in hands-on surgical training since the experience gained from computational simulation is incomparable to actually operating on a model. Moreover, research on the use of MIF in surgery is limited compared to the vast 3D printing research pool to prove the advantages mentioned above.

## Limitations and future scope

Although the application of 3D-printed models was considered beneficial in this study, certain limitations of the study should be noted. First, the study enrolled a small number of patients, and there was no control group to compare the validity of the results. Second, the included patients primarily presented with middle skull base communicating tumors, when ideally, a multi-centered study enrolling patients with different tumor types is warranted to observe for any differences in the outcomes of using 3D-printed models in different groups. Finally, the time spent on printing a workable 3D model is still too long. Considering the time spend on arranging MRI, CT, and CTA examinations, processing radiological scans into a stereolithography file, printing the 3D model (5–8 hours), and finally arranging the surgical operation, the operation might be postponed for 1-2 weeks. This is clearly not suitable for emergencies. Further studies are necessary to address these limitations for popularizing the use of 3D-printed models in the field of surgery.

## Conclusion

3D-printed models showed good structural accuracy and were potentially beneficial for resident education. These models may serve as a helpful assisting method in the treatment of intracranial and extracranial communicating tumors. However, restricted by the limited case number, the true applicability of these models awaits further research to be demonstrated.

### Supplementary Information

**Additional file 1: Supplementary figures and table.**

**Additional file 2: Movie 1.** A short video introducing the 3D-printed model of case seven and the surgical approach adopted.

**Additional file 3: Movie 2.** A short video introducing the 3D-printed model of case two and the surgical approach adopted.

## Data Availability

Data available upon request due to privacy and ethical restrictions.

## Abbreviations

3D: Three-dimensional
SLA: Stereolithography
MRI: Magnetic resonance imaging
CT: Computed tomography
CTA: Computed tomography angiography
MIF: Multimodal image fusion
