# Supplementary material for: Clinical application of a three-dimensional-printed model in the treatment of intracranial and extracranial communicating tumors: a pilot study
Source: 3D Print Med. 2024 Jan 22;10:2. doi: 10.1186/s41205-024-00202-5 (PMC10802061; doi:10.1186/s41205-024-00202-5)
Supplement: Supplementary file 1 — Additional file 1: Supplementary figures and table. [file 41205_2024_202_MOESM1_ESM.docx]

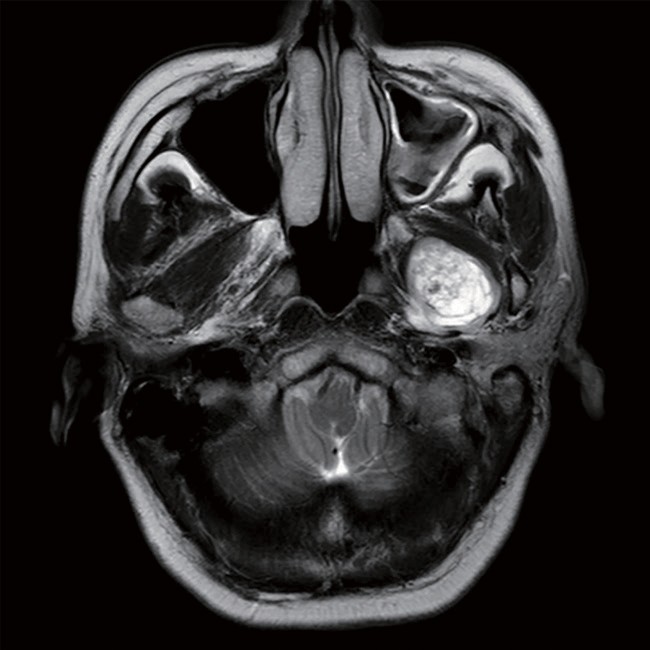


A


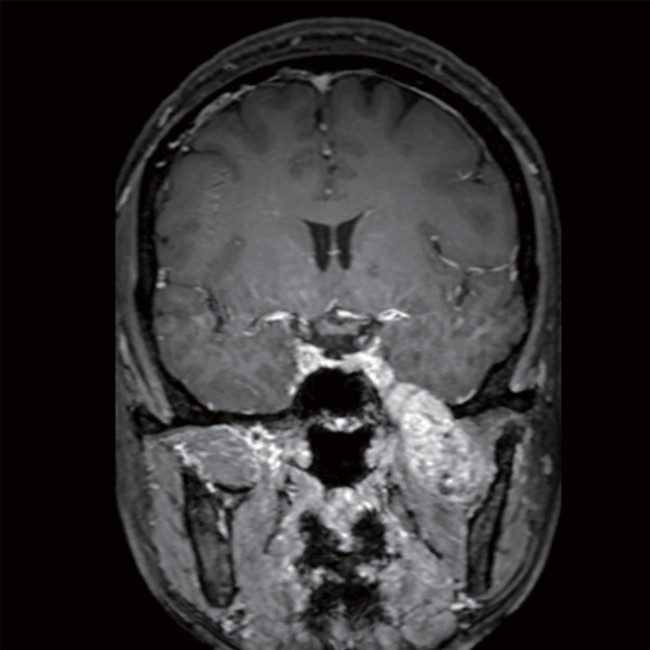


B


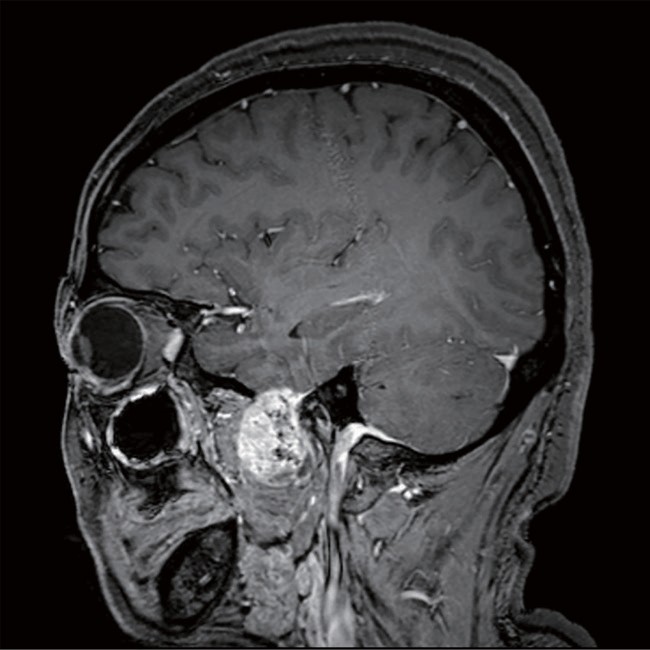


C


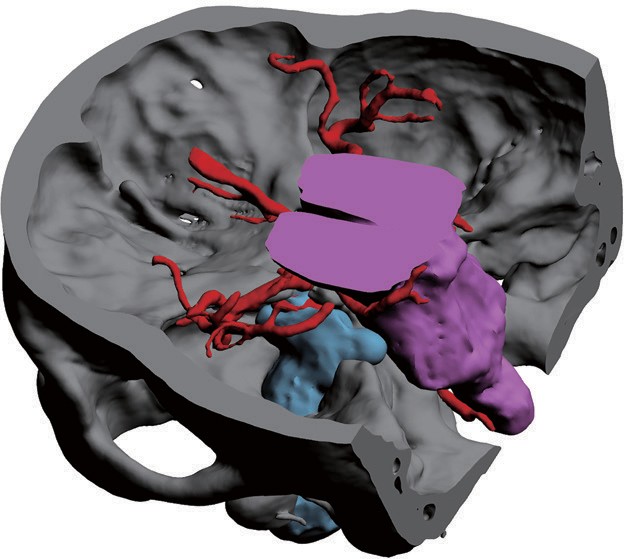

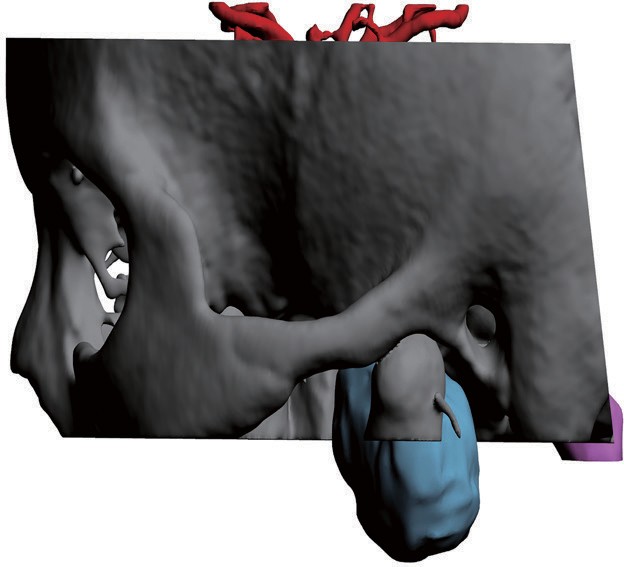
E G


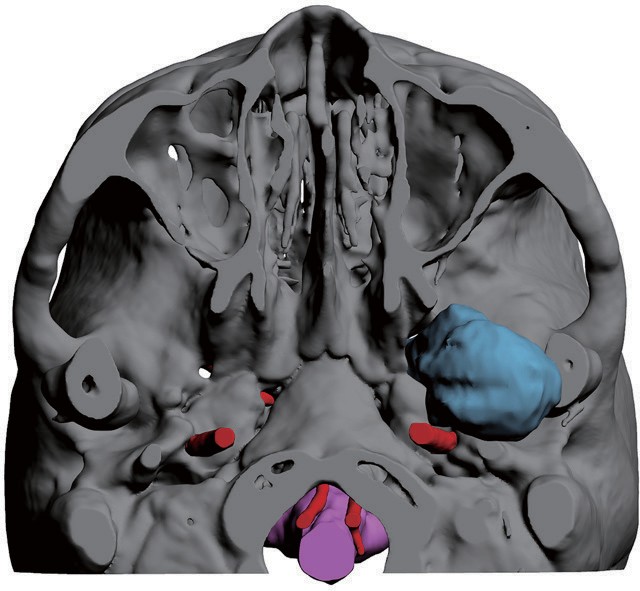


F


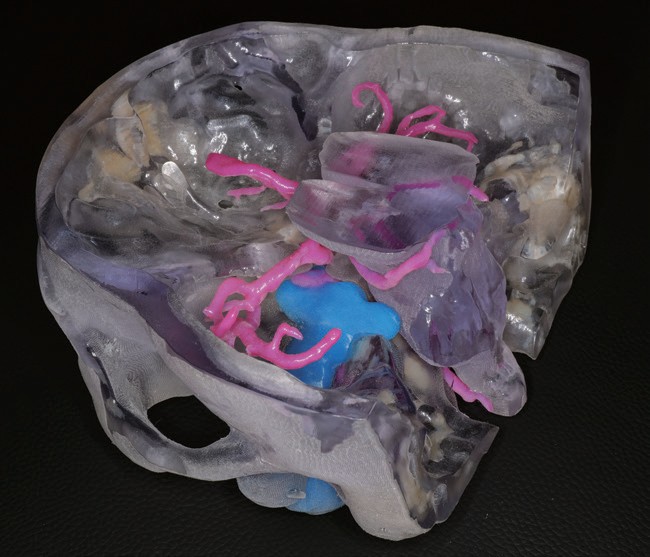


G


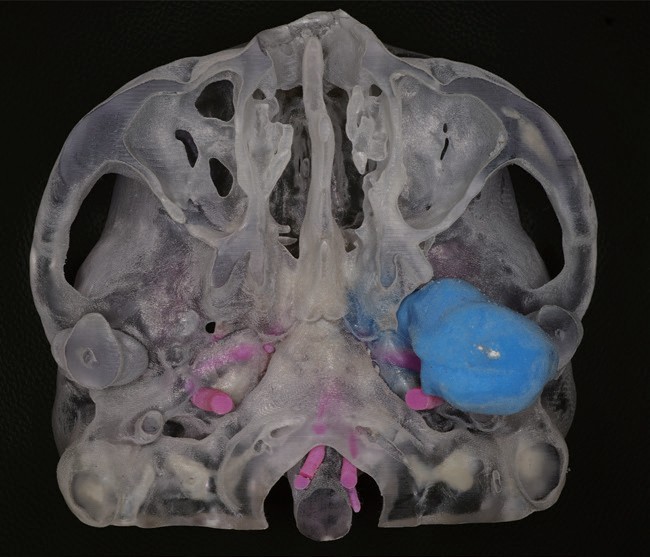


H


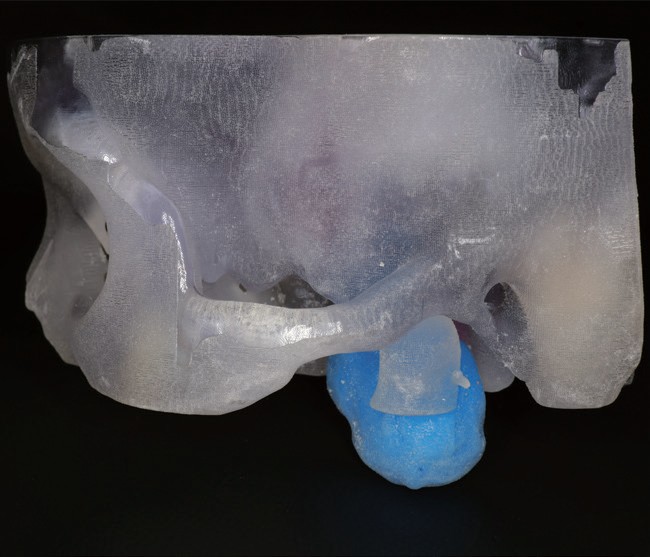


I

**Figure S1** Demonstration of one 3D-printed model (case 9). (A-C) MRI (T1 weighted) images of the patient; (D-F) 3D reconstruction of the tumor and surrounding tissue; (G-I) the printed 3D model.


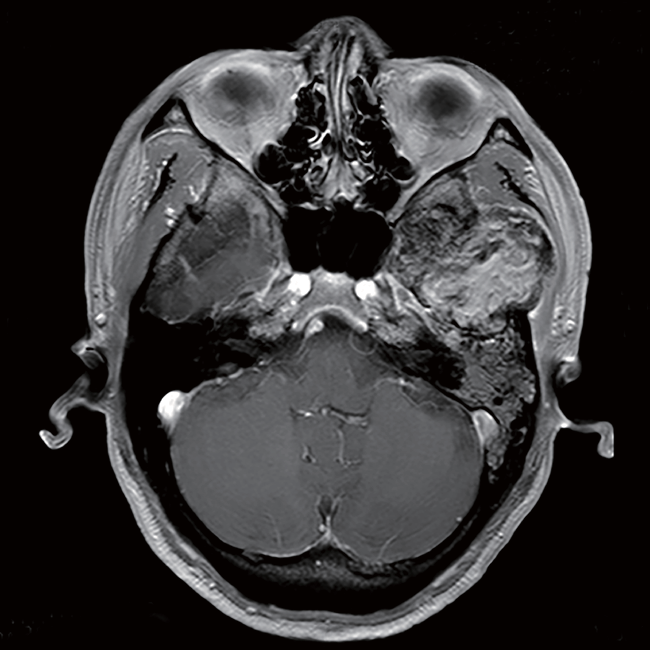


A


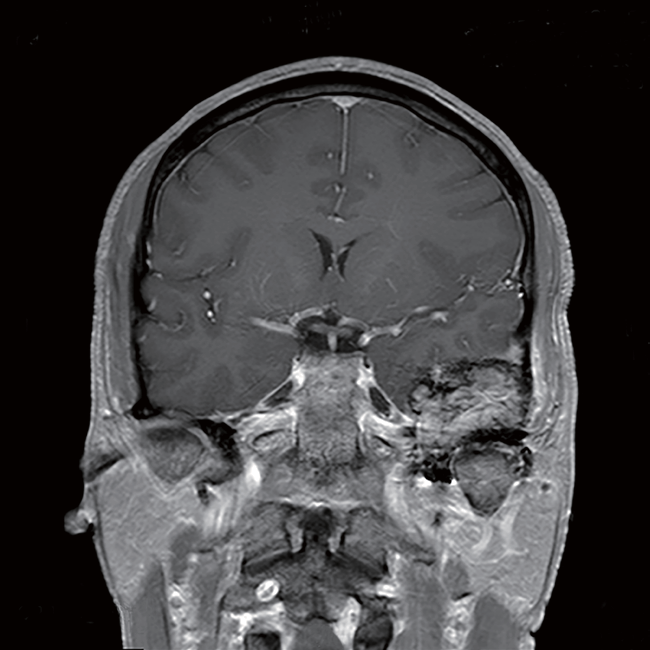


B


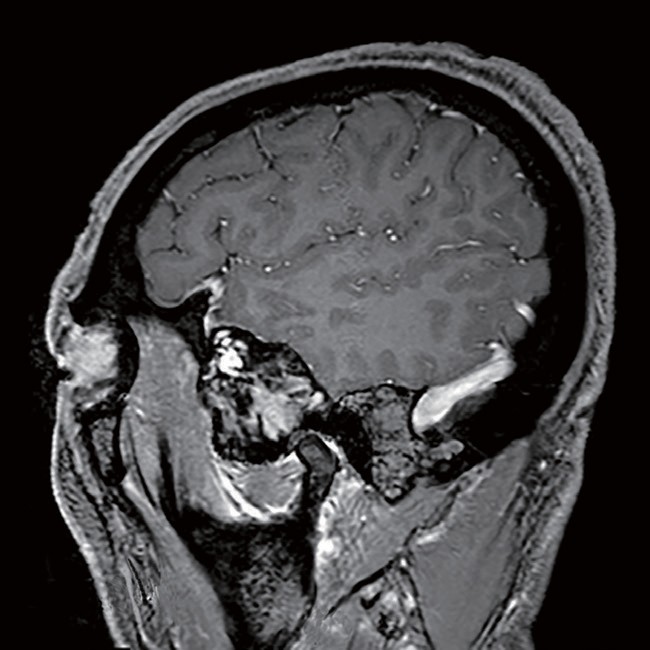


C


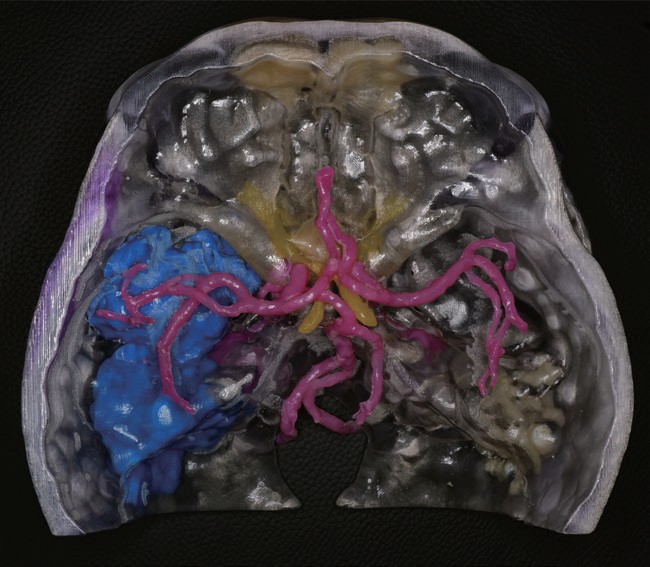


E


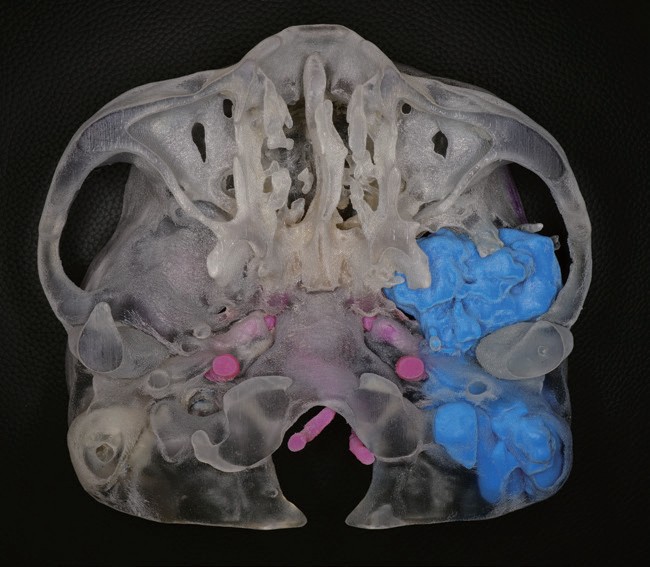


F


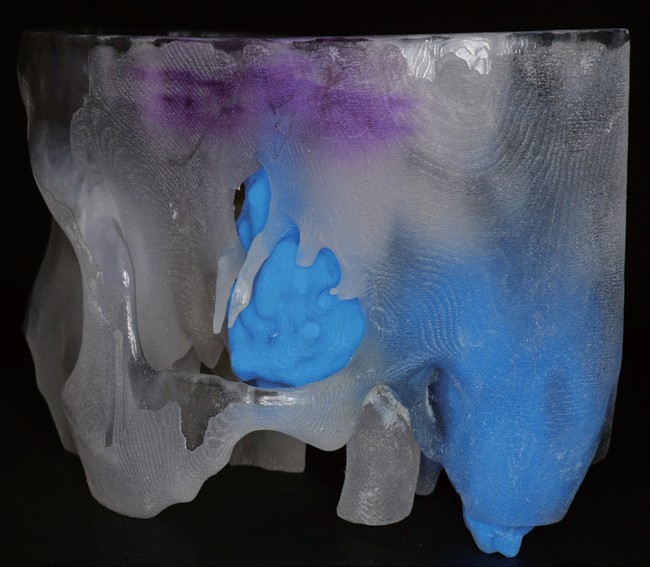


G

**Figure S2** Demonstration of one 3D-printed model (case 2). (A-C) MRI (T1 weighted) images of the patient; (D-F) the printed 3D model.

| **Table S1. Clinicopathological features of all included patients.** | | |
| --- | --- | --- |
| **Variables** | **Value** | **Percentage (%)** |
| **Mean age in years (range)** | 46.5 (29-78) | 100 |
| **Sex** |  |  |
| **Male** | 3 | 37.5 |
| **Female** | 5 | 62.5 |
| **Skull base involvement** |  |  |
| **Anterior**^†^ | 1 | 12.5 |
| **Middle** | 7 | 87.5 |
| **Posterior** | 1 | 12.5 |
| **Tumor size (cm)** |  |  |
| **≥5** | 6 | 75 |
| **<5** | 2 | 25 |
| **Pathological diagnosis** |  |  |
| **Meningioma** | 2 | 25 |
| **Others** | 6 | 75 |
| **Complication** |  |  |
| **Yes** | 1 | 12.5 |
| **No** | 7 | 87.5 |
| **Mean operation time in hours (range)** | 7.55 (3.50-12.75) | 100 |
| **Mean blood loss in milliliters (range)** | 750 (100-2800) | 100 |
| **Mean length of stays in days (range)** | 25 (20-35) | 100 |
| ^†^One case involved both anterior and middle skull base. | | |
